# Does perspective matter? A case study comparing Eulerian and Lagrangian estimates of common murre (*Uria aalge*) distributions

**DOI:** 10.1002/ece3.5083

**Published:** 2019-03-26

**Authors:** Elizabeth M. Phillips, John K. Horne, Jeannette E. Zamon, Jonathan J. Felis, Josh Adams

**Affiliations:** ^1^ School of Aquatic and Fisheries Sciences University of Washington Seattle Washington; ^2^ NOAA Fisheries Northwest Fisheries Science Center Seattle Washington; ^3^ NOAA Fisheries Northwest Fisheries Science Center Hammond Oregon; ^4^ U.S. Geological Survey Western Ecological Research Center Santa Cruz California

**Keywords:** aerial survey, common murre, satellite telemetry, ship‐based survey, species distribution, survey design

## Abstract

Studies estimating species' distributions require information about animal locations in space and time. Location data can be collected using surveys within a predetermined frame of reference (i.e., Eulerian sampling) or from animal‐borne tracking devices (i.e., Lagrangian sampling). Integration of observations obtained from Eulerian and Lagrangian perspectives can provide insights into animal movement and habitat use. However, contemporaneous data from both perspectives are rarely available, making examination of biases associated with each sampling approach difficult. We compared distributions of a mobile seabird observed concurrently from ship, aerial, and satellite tag surveys during May, June, and July 2012 in the northern California Current. We calculated utilization distributions to quantify and compare variability in common murre (*Uria aalge*) space use and examine how sampling perspective and platform influence observed patterns. Spatial distributions of murres were similar in May, regardless of sampling perspective. Greatest densities occurred in coastal waters off southern Washington and northern Oregon, near large murre colonies and the mouth of the Columbia River. Density distributions of murres estimated from ship and aerial surveys in June and July were similar to those observed in May, whereas distributions of satellite‐tagged murres in June and July indicated northward movement into British Columbia, Canada, resulting in different patterns observed from Eulerian and Lagrangian perspectives. These results suggest that the population of murres observed in the northern California Current during spring and summer includes relatively stationary individuals attending breeding colonies and nonstationary, vagile adults and subadults. Given the expected growth of telemetry studies and advances in survey technology (e.g., unmanned aerial systems), these results highlight the importance of considering methodological approaches, spatial extent, and synopticity of distribution data sets prior to integrating data from different sampling perspectives.

## 1 INTRODUCTION

Distribution and abundance data of mobile species are useful for identifying important foraging, migration, and breeding habitats (Elith & Leathwick, 2009; MacArthur, 1972). Data can be obtained from observations collected during surveys within a predetermined frame of reference (i.e., Eulerian sampling) or by sampling discrete locations estimated using animal‐borne tracking devices (i.e., Lagrangian sampling; Rutz & Hays, 2009, Tremblay et al., 2009).

Eulerian survey designs sample at *x*–*y* coordinates at predetermined stations or along contiguous transects, often replicated through time. The primary objective of Eulerian sampling approaches is to obtain information about animal distribution and abundance in a predefined area and time period. In the ocean, vessel‐based Eulerian surveys regularly use direct sightings to quantify the distributions of marine mammals (Ainley, Dugger, Toniolo, & Gaffney, 2007; Ballance & Pitman, 1998; Keiper, Ainley, Allen, & Harvey, 2005) and seabirds (Ainley et al., 2005; Ballance, Pitman, & Reilly, 1997). Ships can survey coastal and offshore ecosystems for relatively long (i.e., weeks to months) periods across hundreds to thousands of kilometers, and simultaneously sample in situ abiotic and biotic factors including seawater temperature, chlorophyll concentration, and prey species abundance and composition, which allows quantification of animal–habitat relationships (Ainley, Ribic, & Woehler, 2012; Fiedler et al., 1998). However, ships are slow relative to the movement of mobile species including seabirds, and the flux of birds into or out of a survey area, as well as vessel avoidance or attraction by some species, may bias distribution and abundance estimates by convoluting spatial patterns with the passage of time (van Franeker, 1994; Wahl & Heinemann, 1979). Aerial surveys (e.g., airplanes and drones) are another Eulerian sampling approach that sample along transects in a relatively short (i.e., hours to days) period and, because the movement of seabirds is slow relative to an aircraft, provide a synoptic estimate of species distribution and abundance (Briggs, Tyler, & Lewis, 1985a; Buckland et al., 2001; Certain & Bretagnolle, 2008). Aircraft can survey areas often inaccessible to ships (e.g., nearshore shallow habitats and ice fields), but may not be able to transit as far offshore to survey pelagic habitats beyond the continental shelf (Henkel, Ford, Tyler, & Davis, 2007; Hodgson, Baylis, Mott, Herrod, & Clarke, 2016). Accordingly, ship‐based and aerial survey data are limited by the spatial and temporal extent and sampling resolution of the survey (Watanuki et al., 2016). Species detectability can also be an issue, as smaller, rare, or cryptic species may not be accurately represented in a data set (Barbraud & Thiebot, 2009; Monk, 2014). Further, for many species, breeding status, sex, and age of individual seabirds cannot be discerned from sighting data, constraining most analyses to the population level. Despite these limitations, transect surveys from ships transiting the world's oceans were an early and significant contributor to studies of pelagic seabird distributions (Brown, 1980; Murphy, 1936; Wynne‐Edwards, 1935), and ship and aircraft surveys continue to be an important component of seabird research (Ainley et al., 2009; Certain & Bretagnolle, 2008; Hunt et al., 2018).

In contrast to Eulerian approaches, Lagrangian survey designs track seabirds through space and time using data logging or tracking devices attached to individuals (Burger & Shaffer, 2008; Hart & Hyrenbach, 2009; Hooker, Biuw, McConnell, Miller, & Sparling, 2007). Satellite‐linked tags that provide near‐real‐time, continuous, and independent sampling are a common tool for Lagrangian sampling (Adams, MacLeod, Suryan, Hyrenbach, & Harvey, 2012; Hatch, Meyers, Mulcahy, & Douglas, 2000). Depending on mobility of the species, a Lagrangian sampling approach may increase the spatial extent and resolution of the survey area compared with an Eulerian perspective (Block, Costa, Boehlert, & Kochevar, 2002). Fine‐scale (i.e., 1–10 km) movements of individuals can be measured with satellite tags and then matched as closely as possible to remotely sensed, modeled, or in situ environmental data to gain insights on correlations between movement and habitat use (Adams & Flora, 2010; Phillips, Horne, Adams, & Zamon, 2018). Further, many tracking devices now carry additional sensors that provide insight on physiology and foraging behavior (Burger & Shaffer, 2008; Ropert‐Coudert & Wilson, 2005; Wilson et al., 2002). While telemetry provides high‐resolution data at an individual level, transmitter cost and logistical challenges can limit the number of tags deployed (i.e., sample size; Lindberg & Walker, 2007). Individual heterogeneity, often attributed to sex and age differences (Gutowsky, Leonard, Conners, Shaffer, & Jonsen, 2015; Hedd, Montevecchi, Phillips, & Fifield, 2014), complicates population‐level inferences (Krietsch et al., 2017), and presence‐only data often require additional steps to develop habitat models (Lobo, Jiménez‐Valverde, & Hortal, 2010; Phillips et al., 2009). Despite sampling constraints, significant advances in tracking technology during the last two decades have resulted in important insights into seabird movement and distribution (Shaffer et al., 2006; Votier, Bicknell, Cox, Scales, & Patrick, 2013; Weimerskirch, Bishop, Jeanniard‐du‐Dot, Prudor, & Sachs, 2016) and tracking tags are now used on many wide‐ranging avian species (Hart & Hyrenbach, 2009; Tremblay et al., 2009).

As the number of Eulerian and Lagrangian studies of marine mammals and seabirds increases (Block et al., 2016; Drew, Piatt, & Renner, 2015), efforts to combine data from these two perspectives have increased. This is due in part to the potential to expand spatial and temporal sampling scales, which could enhance studies of species' distributions and inform conservation efforts (Fujioka et al., 2014; Watanuki et al., 2016). Data from Eulerian and Lagrangian perspectives or platforms may be complementary, but integration can be complicated by biases inherent in data collected from different sampling approaches, including a mismatch in spatiotemporal sampling coverage. Concurrent and spatially overlapping data from both Eulerian and Lagrangian perspectives are rare, consequently differences in species distribution patterns attributable to sampling perspective are difficult to evaluate.

We used contemporaneous data from Eulerian and Lagrangian surveys to examine whether sampling perspective or platform influences estimates of a seabird's distribution. We quantified and compared common murre (*Uria aalge*) density distributions observed during May, June, and July 2012 from ship, aerial, and satellite telemetry surveys in the northern California Current. Murres are one of the most numerous seabird species along the west coast of North America (Briggs, Tyler, Lewis, & Carlson, 1987; Carter et al., 2001; Thomas & Lyons, 2017), with ~532,000 individuals attending colonies and breeding along the Oregon and Washington coasts during spring and summer (April–August; Naughton, Pitkin, Lowe, So, & Strong, 2007; Speich & Wahl, 1989). Nesting adult murres are central place foragers that search for prey within ~100 km of their colony (Davoren, Montevecchi, & Anderson, 2003; Decker & Hunt, 1996; Hatch et al., 2000). Thus, the expected movement constraints of murres and the availability of concurrent ship, plane, and telemetry data sets allowed us to compare spatial patterns of murres observed during the breeding season using different sampling perspectives and platforms.

## 2 METHODS

All sampling was conducted in continental shelf waters along the northern Oregon and Washington coasts, with a focus near the mouth of the Columbia River and colonies adjacent to this geographic feature.

### 2.1 Eulerian sampling

#### 2.1.1 Ship‐based surveys

**Figure 1:**
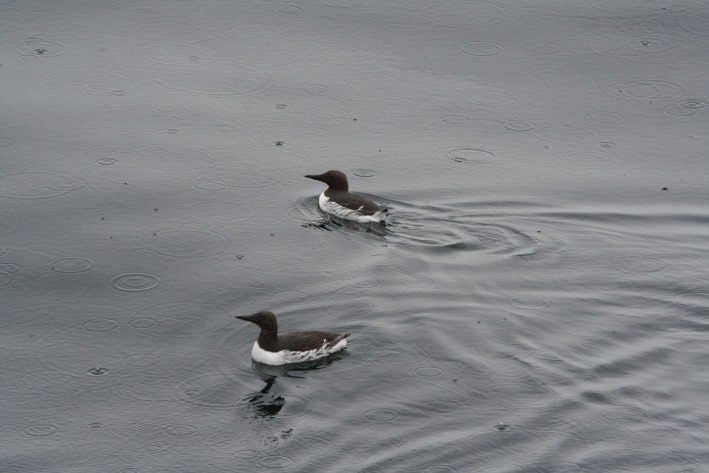
Common murres (*Uria aalge*) observed floating on the surface of the water from a ship survey. Photograph credit: J.E. Zamon/NOAA Fisheries

**Figure 2:**
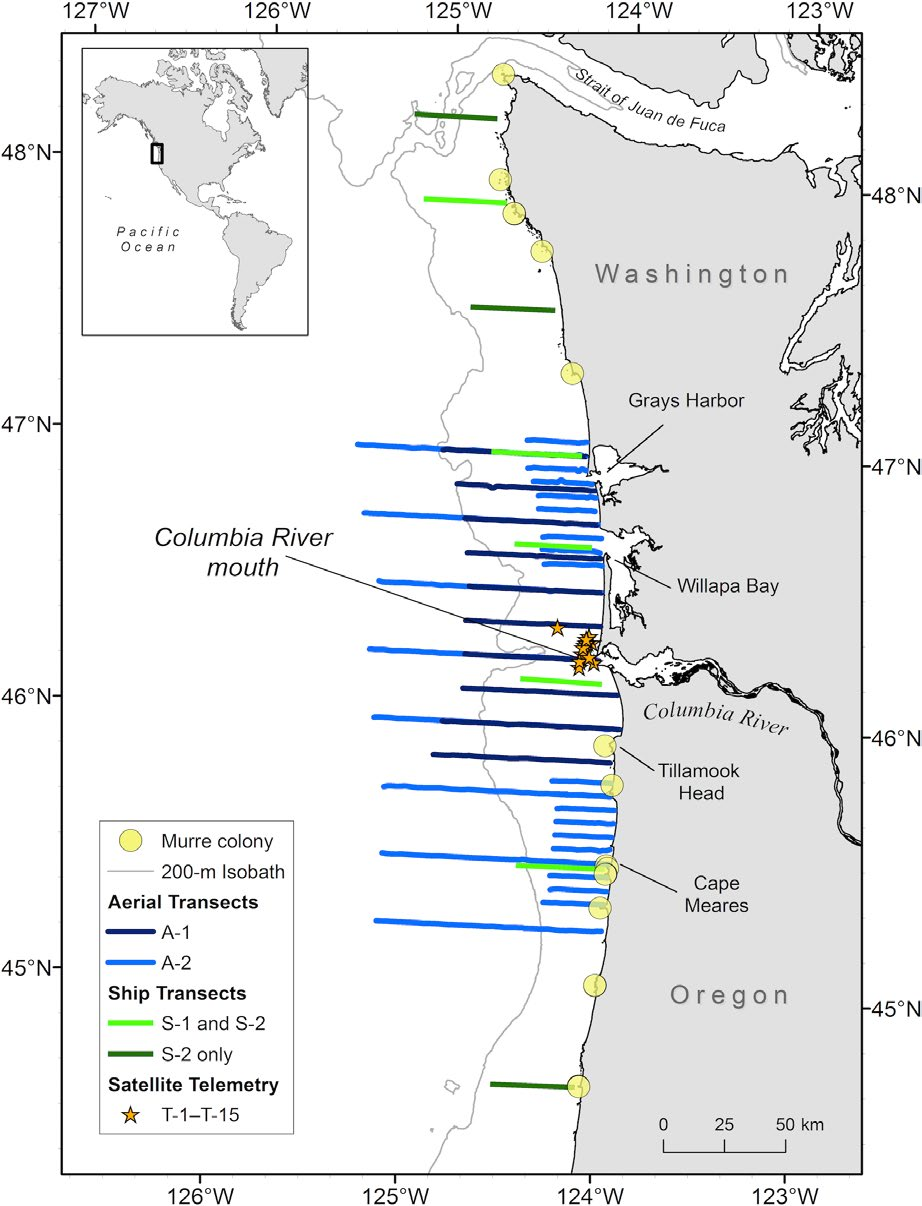
Study area off the Washington and Oregon coast, with geographical points of interest labeled. All surveys were conducted during spring–summer 2012. Ship transects surveyed during 30 May–3 June (S‐1) and 21–28 June (S‐2) are shown in light green; transects surveyed only during S‐2 are shown in dark green. Aerial transects flown on 19 May (A‐1) are shown in dark blue; broad survey transects and focal‐area surveys flown on 1 and 4 July (A‐2) are shown in light blue. Locations of tag deployments for satellite‐tracked common murres (*Uria aalge*; T‐1–T‐15) released near the mouth of the Columbia River on 4–5 May are shown as orange stars. Major murre colonies (>2,000 birds) are shown as yellow points, and those identified in the text are labeled

We used ship‐based data from an ongoing ecosystem research program examining the ocean ecology of salmon off the Washington and Oregon coasts (Brodeur, Myers, & Helle, 2003). Using standard strip transect survey methods (Tasker, Jones, Dixon, & Blake, 1984) during daylight hours in May and June 2012, we collected direct sightings of flying or floating murres (Figure 1) within 300 m of a chartered, commercial fishing vessel (for full details, see Phillips, Horne, & Zamon, 2017). Each sighting was spatially and temporally indexed with Global Positioning System (GPS) coordinates using SeeBird software (v 4.1.5.0; NOAA Fisheries Southwest Fisheries Science Center, La Jolla, California, USA). Each east‐west transect was ~40 km in length, with survey efforts beginning offshore and the ship traveling shoreward for 2 hr at ~5 m/s to within ~3–5 km of shore (Figure 2). To sample a large latitudinal range (44.7–48.2°N) of the northern California Current, the north–south distance between transects ranged from 35 to 90 km. Data were collected along five transects during a survey in late May to early June 2012 (S‐1) and on eight transects during late June 2012 (S‐2; Table 1).

**Table 1:** Description of ship, plane, and satellite telemetry‐based data collections for common murres (*Uria aalge*) in 2012 including sampling perspective and platform type, survey identity, date range, duration, track length, and total sightings or tag locations used for analyses

Perspective	Platform	ID	Date range	Duration (days)	Track length (km)	Total sightings/locations
Eulerian	Ship	S‐1	5/30–6/3	5	145.8	428
		S‐2	6/21–6/28	8	262.1	749
	Plane	A‐1	5/19	1	600.6	618
		A‐2	7/1, 7/4	2	1,160.2	880
Lagrangian	PTT	T‐1	5/5–6/11	37	1541.6	182
		T‐2	5/5–5/23	19	859.3	112
		T‐3	5/6–7/15	70	4,649.3	317
		T‐4	5/5–6/3	29	1,478.3	156
		T‐5	5/5–7/10	66	2,677.6	281
		T‐6	5/5–7/17	72	3,323.5	348
		T‐7	5/5–7/9	65	2,501.0	301
		T‐8	5/5 –7/13	68	3,290.1	337
		T‐9	5/6–7/4	60	2,162.9	295
		T‐12	5/5–5/23	18	670.4	100
		T‐13	5/6–7/23	79	4,409.6	341
		T‐15	5/5–7/11	67	2,890.4	317

#### 2.1.2 Aerial surveys

We used data from aerial surveys of the northern California Current conducted by the U.S. Geological Survey (Adams, Felis, Mason, & Takekawa, 2014). Sightings of murres were recorded from twin‐engine, high‐wing aircraft (Partenavia P‐68, Aspen Helicopters, Oxnard, CA, or Commander AC‐500, GoldAero, Arlington, WA) along predetermined, systematic, east‐west‐oriented transects flown at 160 km/h from the 2000‐m isobath to shore (~90 km; Figure 2). Using aerial survey methods, modified slightly from Mason et al. (2007), two observers counted all birds observed in 150‐m strip transects (75 m per side) from 60 m above sea level. The low‐elevation survey methods were reviewed by NOAA's National Marine Fisheries Service, who granted a Letter of Concurrence to the U.S. Geological Survey. The number and location of individual murres were linked using observation time with GPS data that allowed simultaneous collection of coordinates. Sampling occurred in a latitudinal range from 45.2 to 47.0°N. Data were collected on 10 transects on 19 May 2012 (A‐1) and on 24 transects on 1 and 4 July 2012 (A‐2; Table 1). Transects flown during A‐1 were spaced 13.9 km apart and extended 72.4‐km offshore, whereas A‐2 included a mix of broad survey transects (27.8‐km spacing, up to 93.6‐km offshore) and two focal‐area surveys (each with ten, 25‐km‐long parallel transect lines spaced 6 km apart) nested within the broad survey transects (Adams et al., 2014; Figure 2). For this study, we treated counts of murres obtained during the two July surveys as one survey for analyses (i.e., all transects were analyzed together) unless otherwise noted.

### 2.2 Lagrangian sampling

#### 2.2.1 Satellite telemetry

We used locations collected from satellite tags (Telonics TAV‐2617 platform terminal transmitters [PTTs]) deployed on 12 murres captured and released at night at sea near the mouth of the Columbia River on 4 and 5 May 2012 (Figure 2). Authority for satellite telemetry was provided by USGS Bird Banding Laboratory Auxiliary Marking Authority no. 22911 (J.A.) and no. 23682 (J.E.Z.), and State of Washington Scientific Collection Permit no. 05‐500 (J.E.Z.). Capture and tagging methods were approved under the USGS Animal Care and Use Committee #WERC‐2007–03. PTTs were programmed to transmit every 60 s for 4 hr in the morning (08:00–12:00 hours) and 4 hr in the evening (14:00–18:00 hours), which coincided with Eulerian surveys that were conducted during daylight hours. Locations of individual birds were determined using the ARGOS system (www.argos-system.org; CLS, 2013) and archived via the Satellite Tracking and Analysis Tool (STAT; Coyne & Godley, 2005). To resolve tag attachment or instrument failure, we removed data from tags that did not transmit for more than 2 weeks, had intermittent transmissions (e.g., 5‐day gap in transmissions), or showed evidence of halted movement (i.e., when median daily movements fell below the 95% confidence interval of average movement of birds for the sampling year; S. Loredo pers. comm.). To maximize location accuracy, all ARGOS location class data (LC‐3 through LC‐B, excluding LC‐Z) were filtered using speed, distance, and angle, resulting in a nominal spatial accuracy of 3 km (*mfilter* function in R package *argosfilter*, Freitas, Lydersen, Fedak, & Kovacs, 2008; for full details see Phillips et al., 2018). We also plotted all tag locations in ArcMap 10.3 (ESRI, Redlands, CA) over a high‐resolution land layer to determine whether any tagged murres utilized colonies during the study period. To determine sex of tagged birds, we collected blood from each murre during tag deployments by aseptic puncture of the medial metatarsal vein and placed one drop of blood on a buffered molecular sexing card for analysis by Zoogen, Inc. (Davis, CA).

### 2.3 Data analysis

For the two Eulerian data sets, we first compared overall density of murres observed during ship and aerial surveys. We calculated densities of murres observed during ship‐based surveys by dividing the total number of murres counted in 3‐km bins (~10‐min increments) by the strip area searched (0.9 km^2^) to obtain murres/km^2^. Similarly, we calculated densities of murres observed during aerial surveys by dividing the total number of murres counted in 2.4‐km bins (~1‐min increments) by the strip area (either 0.18 km^2^ [one observer] or 0.36 km^2^ [two observers]) to obtain murres/km^2^. To determine whether mean densities differed within data sets, we compared densities observed during S‐1 and S‐2, and A‐1 and A‐2 using *t* tests (Zar, 1999). To determine whether offshore distribution patterns varied by survey method, we evaluated histograms of the frequency of murres observed as a function of distance from shore. We removed the focal‐area survey data from S‐2 histogram plots as these transects did not extend beyond 25 km of shore.

Because absolute densities cannot be estimated from locations of satellite‐tagged murres, we calculated Brownian bridge utilization distributions (Horne, Garton, Krone, & Lewis, 2007) to estimate each tagged murre's probability of occurrence using the *kernelbb* function in R package *adehabitat* (Calenge, 2006). A utilization distribution (UD) is a probability distribution that gives the probability density that an animal is found at a given point in space. It is estimated by sampling the location of individuals in space through time. The Brownian bridge UD approach provides an estimate of space use from animal trajectories with serial autocorrelation of relocations (Horne et al., 2007). We created an overall 99% UD for all 12 murres by first calculating 99% UDs for each individual bird (i.e., 99% cumulative probability that an individual murre would be present in all 3‐km^2^ cells) and then proportionately weighting the individual UD by its tracking duration (i.e., tracking days per individual divided by total tracking days for all individuals) and summing with the rest of the individually weighted UDs. The overall UD represents a summed probability density surface of tagged murre space use during the full duration of tag transmissions, with a spatial resolution of 3 km^2^. Because UD values are calculated from a population of individuals and have a spatial context, they are similar to mapped densities and can be compared. To estimate concurrent tagged murre distributions during each ship or aerial survey, we calculated separate UDs of tagged murres during each survey time period, using the full spatial extent of tag locations. For the ship surveys, this included a UD during 30 May–3 June (S‐1; *n* = 10 tagged birds, *n* = 233 locations) and 21 June–28 June (S‐2; *n* = 8 tagged birds, *n* = 298 locations). To compare with the aerial surveys, we calculated a UD on 19 May (A‐1; *n* = 12 tagged birds, *n* = 60 locations) and on 1 and 4 July (A‐2; *n* = 8 tagged birds, *n* = 157 locations). Because telemetry data were available for the periods before, between, and after each ship or aerial survey, we calculated separate UDs during these periods to determine whether tagged murre distributions were different earlier or later in the season when Eulerian survey data were unavailable. Finally, the distance from shore of satellite‐tagged murre locations was tabulated and plotted to compare with offshore distributions of murres observed during ship and aerial surveys.

To compare distributions of murres observed from ship and aerial surveys with the satellite telemetry‐derived UDs, we created interpolated, continuous‐surface density distributions using the *kernel interpolation with barriers* tool in ArcMap 10.3. Kernel density estimation (KDE) is a simple nonparametric statistical technique that estimates a real‐valued function as the weighted average of neighboring observed data (Worton, 1989). The weight is defined by the kernel, such that closer points are given greater weights, and smoothness is set by the kernel bandwidth (Worton, 1989). We used a first‐order polynomial kernel function and kernel bandwidths set to the minimum north–south distance between transects to create a smooth prediction surface from ship and aerial transect observations. This is similar to approaches used in other studies of seabird distributions derived from transect survey data (O'Brien, Webb, Brewer, & Reid, 2012; Perrow, Harwood, Skeate, Praca, & Eglington, 2015). To quantify similarities in murre spatial distributions among ship, plane, and telemetry data sets, we calculated percent overlap of concurrent kernel density (KD) and UD surfaces using the *tabulate intersection* tool in ArcMap 10.3. This approach calculates the spatial overlap based on the surface area of each predicted distribution. We limited overlap analyses to the area of each KD surface, thereby excluding UD surfaces from tagged birds that extended beyond the area surveyed during each ship or aerial survey. We compared overlap of full (99%) and 50% (i.e., core use areas) UD distributions that occurred within the full and 50% KD during each ship or aerial survey. We also calculated and plotted the geographic mean center, or center of gravity (CG), of satellite‐tagged murre locations and murres observed during each ship and aerial survey (Bez & Rivoirard, 2001; Woillez, Poulard, Rivoirard, Petitgas, & Bez, 2007; Woillez, Rivoirard, & Petitgas, 2009), and measured the Euclidean distance between CGs for each survey comparison.

## 3 RESULTS

### 3.1 Eulerian sampling

#### 3.1.1 Ship‐based surveys

**Figure 3:**
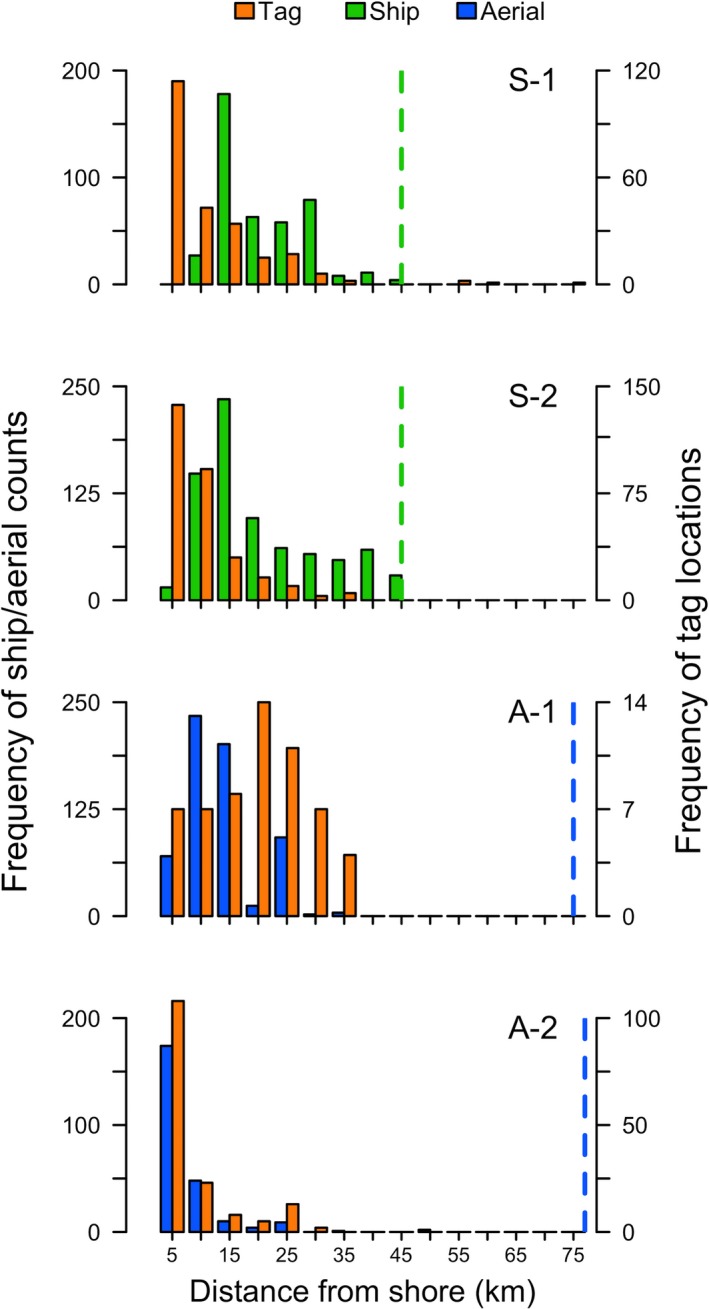
Distribution of common murre (*Uria aalge*) distances from shore during ship (S‐1, S‐2) and broad aerial surveys (A‐1, A‐2), and satellite telemetry tag locations during each ship or aerial survey. Dashed vertical lines indicate the offshore extent of each ship or aerial survey. During A‐2, the plane surveyed 94‐km offshore, but the x‐axis was truncated because no murres were observed more than 75 km from shore

**Figure 4:**
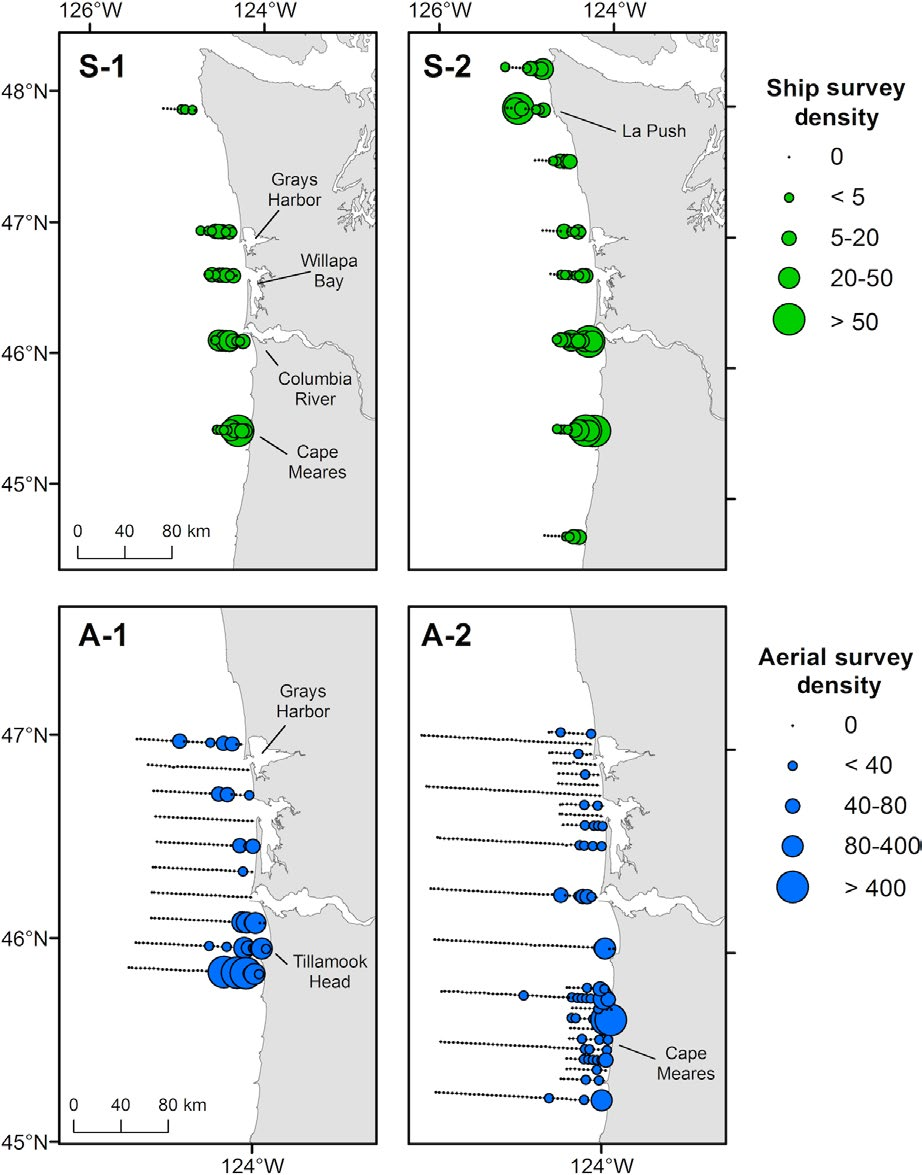
Density distributions of common murres (*Uria aalge*) observed in 2012 during ship surveys (green points; 3‐km bins) on 30 May–3 June (S‐1) and 21–28 June (S‐2), and aerial surveys (blue points; 2.4‐km bins) on 19 May (A‐1) and 1 and 4 July (A‐2) in the northern California Current. Geographic locations identified in the text are labeled

We counted a total of 428 murres during 43.4 km^2^ of survey effort during S‐1 and 749 murres during 78.8 km^2^ of survey effort during S‐2 (Table 1). Murres were found across most of the extent of ship surveys (4.7–44.8 km from shore), with greatest numbers of individuals occurring between 10 and 20 km of shore (Figure 3). Mean densities of murres were not significantly different between S‐1 (9.9 murres/km^2^) and S‐2 (9.5 murres/km^2^; *t*_72.3_ = −0.076, *p* = 0.940). Murre densities were consistently greatest adjacent to a large murre colony on the Cape Meares (CM) transect and on the Columbia River (CR) transect (Figure 4). During S‐1, mean densities of murres on the CM transect (26.0 murres/km^2^) and on the CR transect (13.9 murres/km^2^) were approximately five to nine times greater than the mean density observed on the other three transects (2.8 murres/km^2^). During S‐2, mean densities on the CM (30.0 murres/km^2^) and CR (23.3 murres/km^2^) transects were also greater than the other transects. We calculated intermediate murre densities along the central Washington coast near Grays Harbor and Willapa Bay during S‐1 and S‐2 (2.7–4.2 murres/km^2^). Similar intermediate densities of murres were observed off the northern Washington coast on S‐2 (3.2–3.7 murres/km^2^), with the exception of greater mean densities near La Push (10.2 murres/km^2^; Figure 4). Low densities (mean: <3.0 murres/km^2^) occurred in the southernmost portion of the survey area off the central Oregon coast on S‐2 (Figure 4).

#### 3.1.2 Aerial surveys

We counted a total of 618 murres during 45.1 km^2^ of survey effort during A‐1 and 880 murres during 162.5 km^2^ of survey effort during A‐2 (Table 1). During aerial surveys, we observed murres between 0.3 and 50 km from shore (Figure 3). The offshore distribution of murres during A‐1 was primarily between 5 and 25 km from shore, with greatest numbers of individual murres located 10–15 km from shore. During A‐2, most murres occurred within 5 km of shore. Mean densities did not differ between A‐1 (13.7 murres/km^2^) and A‐2 (5.4 murres/km^2^; *t*_370.7_ = 1.54, *p* = 0.125). During A‐1, densities of murres were greatest on the three transects along the northern Oregon coast (mean: 35.1 murres/km^2^), including the transect adjacent to the Tillamook Head murre colony and near the mouth of the Columbia River (Figure 4). Although we observed lesser densities of murres off the southern Washington coast, relatively greater densities (4.4 murres/km^2^) were observed near Grays Harbor during A‐1. During A‐2, greatest densities (mean: 9.6 murres/km^2^) were also observed on transects off the northern Oregon coast near murre colonies at Tillamook Head and Cape Meares, and least densities (mean: 0.48 murres/km^2^) were observed off the southern Washington coast near Willapa Bay and Grays Harbor (Figure 4).

#### 3.1.3 Lagrangian sampling

**Figure 5:**
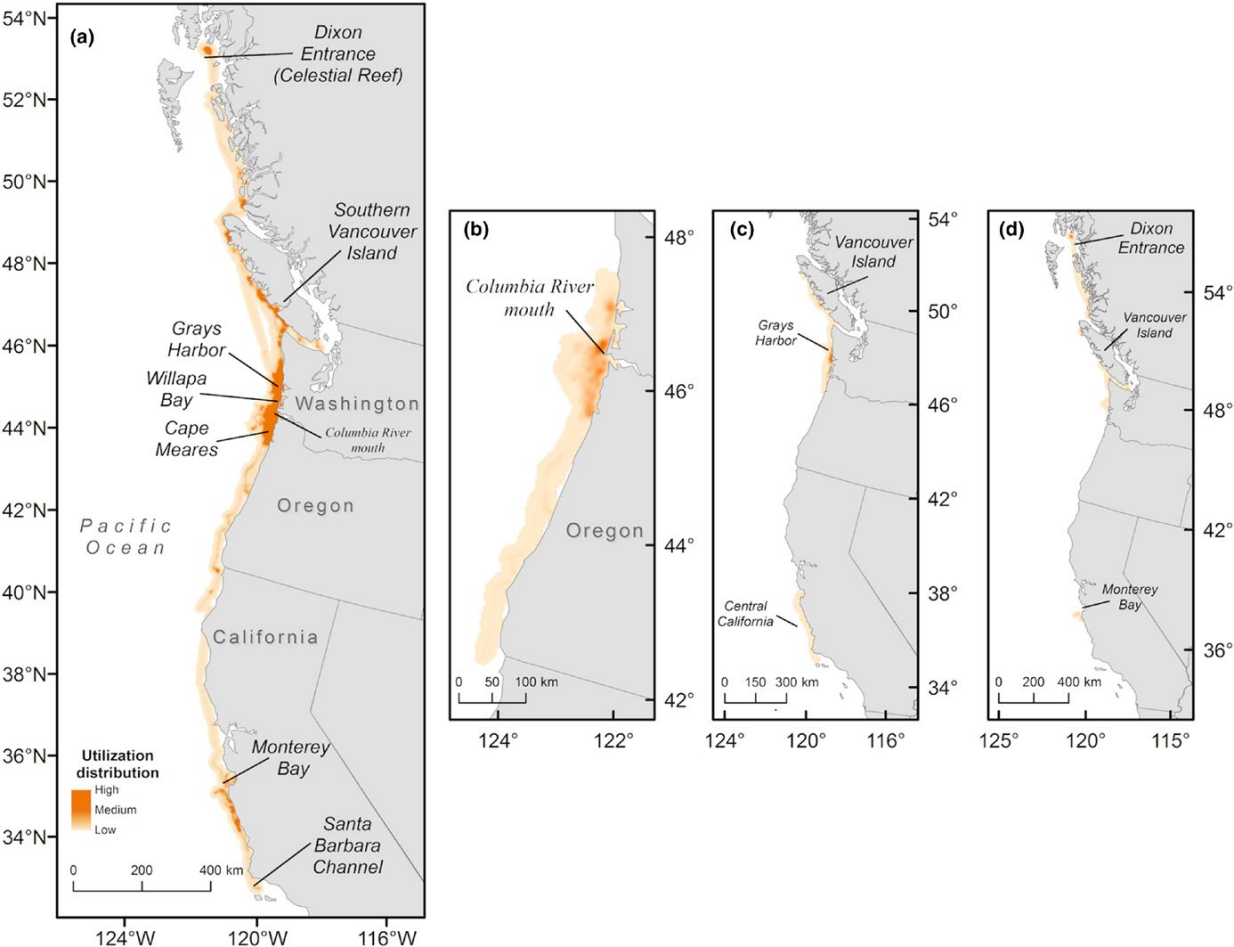
Utilization distribution (99%) of 12 satellite‐tagged common murres (*Uria aalge*) observed in 2012 during (a) the full study period (4 May–23 July), (b) before ship or aerial surveys began (4 May–18 May), (c) between ship surveys (4 June–20 June) and (d) after the second aerial survey (A‐2) was completed (5 July–23 July). Locations of high‐use areas identified in the text are labeled

We tracked satellite‐tagged murres for an average of 54.2 ± 21.9 days (mean ± *SD*) between early May and early July. Tracking duration ranged from 18 to 73 days, with 7 of 12 (58%) tags transmitting for ≥63 days (Table 1). Fifty‐eight percent (*n* = 7) of tagged murres were female, 33% (*n* = 4) were male, and the sex of one murre could not be determined. Most murre locations occurred within 5–10 km from shore (range: 3–76 km), and were closer to shore than murres observed during ship or aerial surveys, except during A‐1 when the number of tag locations was smaller (Figure 3). Tracked murres occupied a vast at‐sea area (114,900,000 km^2^; i.e., >2 times the area of the State of California). Overall, the 99% utilization distribution (UD) indicated a broad latitudinal use of nearshore coastal waters between British Columbia and central California (Figure 5). Highest use areas were located off the northern Oregon and southern Washington coasts, and the west coast of southern Vancouver Island, British Columbia, Canada (Figure 5a). We observed some use of waters near a small colony along the central Washington coast (Grenville Arch), as well as Tillamook Head and Cape Meares in northern Oregon, but obvious central place foraging behavior, such as repeated trips to land, was not observed in the tracking data. One male murre traveled ~1,500 km to southern‐central California and spent most of its time during the study between Monterey Bay and the Santa Barbara Channel; two female murres moved ~950 km north to the west coast of Canada near the southeast Alaska–British Columbia border (i.e., Celestial Reef in Dixon Entrance). Two additional females and one male murre moved into waters along the west coast of Vancouver Island, while the rest of the tagged murres (three females, two males, and one undetermined sex) remained in Washington and Oregon waters for the duration of tag transmissions.

**Figure 6:**
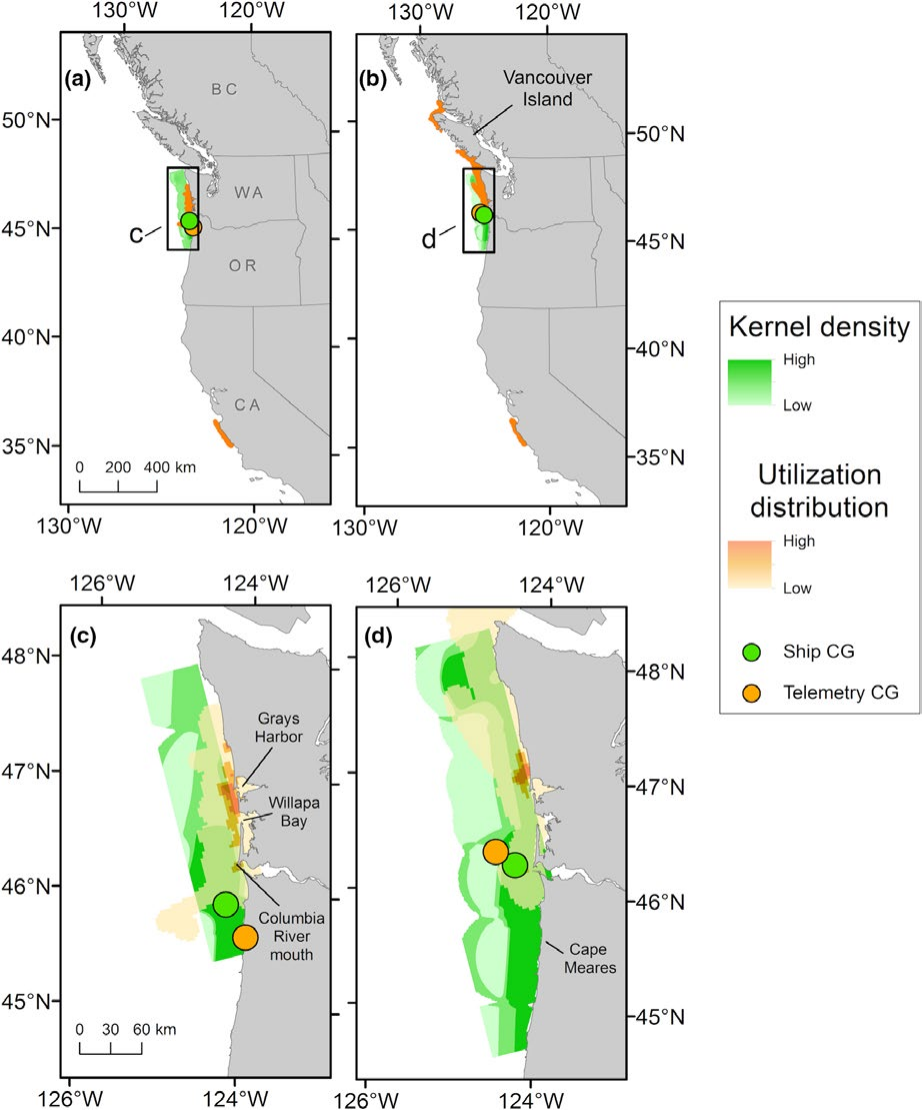
Common murre (*Uria aalge*) density distributions observed during ship surveys (green surface) and satellite telemetry (orange surface) during the same time period in 2012. Distributions of tagged murres observed during S‐1 (30 May–3 June) are shown (a) at the broad scale and (c) within the area surveyed by the ship. Distributions of tagged murres observed during S‐2 (21 June–28 June) are shown (b) at the broad scale and (d) within the area surveyed by the ship. The corresponding geographic centers of gravity (CG) are shown as green or orange points. Locations identified in the text are labeled

The UD of tagged birds calculated for the period before the first Eulerian survey occurred indicated that satellite‐tracked murres exhibited high spatial use of waters along the southern Washington and northern Oregon coast, with a high‐use area near the mouth of the Columbia River (Figure 5b). During this time, one male murre flew south into southern Oregon and northern California waters. With the exception of the murre that moved into California waters, the UD calculated during S‐1 indicated that most tagged murres remained aggregated off Grays Harbor and Willapa Bay, and near the mouth of the Columbia River, similar to observations made during the ship survey (Figure 6a, c). Spatial overlap between the 99% UD and the full kernel density surface (KD) during S‐1 was 35%, and 25% of core use areas (50% UD and KD) overlapped. The geographic mean centers of gravity (CGs) were 37 km apart. A similar spatial distribution of tagged murres was observed during the 16 days between ship surveys S‐1 and S‐2, although the UD revealed that some tagged murres shifted north during this period into Canadian waters along the west coast of Vancouver Island (Figure 5c). During S‐2, tagged murres were more broadly distributed throughout Washington coastal waters, with greatest spatial use near Grays Harbor (Figure 6b, d). Overlap between the 99% UD and the full KD during S‐2 was 30%, and 27% of core use areas overlapped. The CGs were separated by 22 km. Spatial distributions of murres observed during both ship surveys were similar, with most murres observed in northern Oregon waters near Cape Meares; CGs between the two ship surveys were separated by 44 km. After the ship surveys were completed, some tagged murres continued moving north into Canadian waters, with two birds moving as far north as Dixon Entrance (Celestial Reef) near the Alaska–British Columbia, Canada border (Figure 5d). Some murres also remained within Washington coastal waters, although the UD indicated minimal spatial use of this area (Figure 5d).

**Figure 7:**
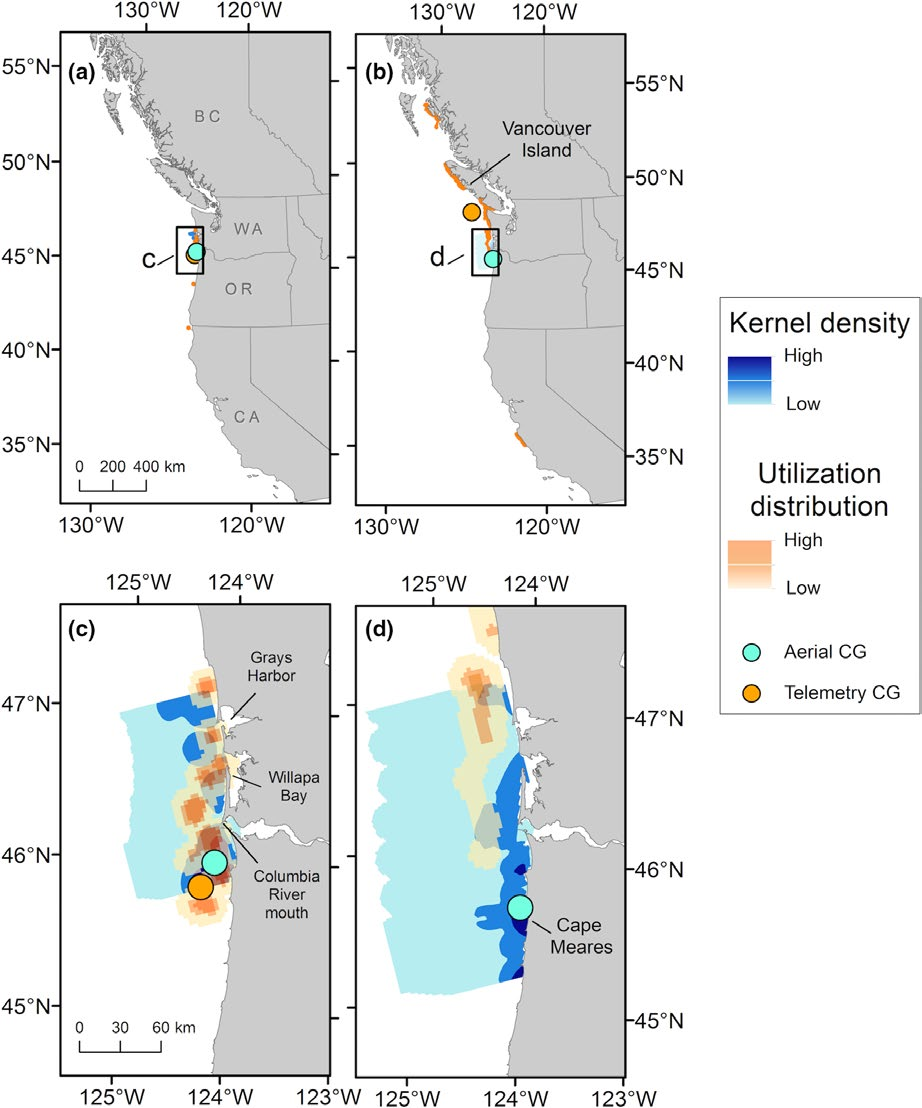
Common murre (*Uria aalge*) density distributions observed during aerial surveys (blue surface) and satellite telemetry (orange surface) during the same time period in 2012. Distributions of tagged murres observed during A‐1 (19 May) are shown (a) at the broad scale, and (c) within the area surveyed by the plane. Distributions of tagged murres observed during A‐2 (1 and 4 July) are shown (b) at the broad scale and (d) within the area surveyed by the plane. The corresponding geographic centers of gravity (CG) are shown as blue or orange points. The CG of tagged murres observed during A‐2 was 302 km north of the CG of murres observed from the plane and only shown on the broad scale map. Geographic locations identified in the text are labeled

The UD during A‐1 indicated that the majority of tagged murres used waters near Grays Harbor, Willapa Bay, and off the mouth of the Columbia River (Figure 7a). During A‐1, 56% of the 99% UD overlapped with the full KD, and 39% of core use areas overlapped. The CGs of murres were separated by 21 km (Figure 7c). Spatial distributions of tagged murres were similar to A‐1 during the 41 days between aerial surveys. However, during A‐2 tagged murres showed high use of waters along the west coast of Vancouver Island and low use of waters near Grays Harbor (Figure 7b). In comparison, murre densities observed from the plane were greatest farther south on the Oregon coast, and overlap between the UD and KD during A‐2 was only 12%, and only 4% of core use areas overlapped (Figure 7d). The distance between the CGs during A‐2 was 302 km, reflecting the northward movement of tagged murres and southerly distribution of murres observed during the aerial survey. In comparison, the CGs of murres observed during A‐1 and A‐2 were only 33 km apart, and located off northern Oregon, similar to the murre CG locations observed during ship surveys. After aerial surveys were completed, locations of tagged murres were widespread in coastal Washington and Canadian waters, primarily along the west coast of southern Vancouver Island and farther north near Dixon Entrance (Celestial Reef; Figure 5d).

## 4 DISCUSSION

We used concurrent data from ships, planes, and satellite telemetry to illustrate that seabird distributions inferred from independent, contemporaneous data sets can indicate similar high‐use areas, but differences in survey perspective and spatiotemporal extent can influence observed patterns. At the spatiotemporal scale of the northern California Current during May 2012, distributions of murres observed in all three data sets were similar and indicated high use of nearshore waters along the Washington coast, near the mouth of the Columbia River, and in northern Oregon near some of the largest murre colonies along the coast, including Cape Meares and Tillamook Head (Carter et al., 2001; Naughton et al., 2007). This is not surprising given that the study period coincided with the breeding season for murres (April–August), a time when both breeding and nonbreeding murres aggregate on the water near colonies before and after foraging bouts (Ainley, Nettleship, Carter, & Storey, 2002; Zador & Piatt, 1999). Regardless of latitude, all murres occurred primarily within 0–25 km of the coast, with tagged murre locations generally occurring closer (3–5 km) to shore than murres observed during ship surveys, which did not survey in shallow water within ~5 km of shore due to hull draft. Aerial surveys revealed nearshore distributions of murres more similar to the telemetry data, particularly during A‐2. Consistently similar densities of murres observed from ship and aerial surveys during May, June, and July demonstrate that large numbers of murres occupy the northern California Current during spring and summer, and that both Eulerian methods can effectively survey the regional distribution of this relatively large‐bodied, coastal seabird (Briggs, Tyler, & Lewis, 1985b; Henkel et al., 2007). Satellite telemetry results during the early part of the study indicated similar spatial distributions of murres across independent data sets, but we documented a broader latitudinal distribution of tagged murres later in the study period as individual birds moved beyond the boundaries of the Eulerian survey transects.

The relatively stable density distributions of murres observed in the ship and aerial survey data contrast with the dynamic distributions observed in the telemetry data and illuminate how different survey perspectives can reveal differing patterns of species' distributions. Estimates of murre distributions observed from all three platforms during May indicated high use of waters in southern Washington and aggregation near colonies in northern Oregon. Relatively high overlap of core utilization distributions and close association of geographic centers of gravity (<40 km apart) suggest that most of the murres observed in each survey data set were collocated in a relatively small region of the northern California Current. One of the highest use areas occurred near the mouth of the Columbia River, which is a productive area that supports a variety of prey fish for seabirds and attracts murres (Litz, Emmett, Bentley, Claiborne, & Barceló, 2013; Phillips et al., 2017). The consistent occurrence of murre aggregations near the mouth of the Columbia River, and the relative ease of capturing murres from the water, is the primary reason that all of the at‐sea captures and tag deployments during the study occurred in this area. There are no active murre colonies along the coast between the mouth of the Columbia River and Grays Harbor, so our results suggest that murres observed in this area were breeding birds that commuted at least 60 km north from large colonies in northern Oregon or moved a minimum of 50–100 km south from colonies along the Washington coast (e.g., Bodelteh Islands, Grenville Arch Rock; Thomas & Lyons, 2017). Alternatively, as the telemetry data suggest, murres observed in this area may not be associated with a colony (i.e., nonbreeders) and therefore able to continually occupy productive waters near the river mouth without returning to coastal colonies.

Although the data from May suggest that common murres in the northern California Current are locally resident, examination of the telemetry data from June and July demonstrates unexpected high mobility among tagged murres, a shift in high‐use areas with time, and greater use of distant coastal waters in California and British Columbia later in the study period. Thus, at least a portion of the murre population occupying the Washington and Oregon coasts are transient, with a predominantly northward flux of individuals occurring between May and July. The ship and aerial surveys may have observed a portion of the murre population that are locally resident from May through July, or new transient individuals that moved into the study area as other murres moved out of the area. While the proportion of murres that are resident or transient is unknown, we conclude that the population of murres occupying the northern California Current likely consists of a mixed group of central place foraging adults and vagile, nonbreeding adults and subadults that differ in their occupancy and use of the California Current. The murres tracked in this study may have been young birds, nonbreeding adults, or failed breeders because these groups exhibit greater dispersals away from colonies than breeding birds (Hatch et al., 2000). Alternatively, the unexpected mobility could indicate that tagging caused individuals to change their movement and/or breeding behavior (see Phillips et al., 2018 for a discussion).

While regional densities of murres observed from ship and aerial surveys were similar during the study period, and the surveys were relatively synchronous, the differences in survey timing and spatial resolution may explain fine‐scale disparities in spatial patterns (van Franeker, 1994; Ronconi & Burger, 2009; Ryan & Cooper, 1989). Ship surveys were designed to sample the entire coast from central Oregon to northern Washington, and transects were separated by 35–90 km, which allowed for observations of murre densities across a wider range of the northern California Current but also may have obscured higher‐resolution variability. In comparison, the aerial surveys were more limited in their overall latitudinal extent but the greater number of more closely spaced transects, especially the focal‐area surveys which were only 6 km apart, may have captured higher‐resolution variability in hourly and daily murre distributions than in ship surveys. Murres are known to aggregate near convergent fronts formed along the boundary between fresh and saltwater near the mouth of the Columbia River (Phillips et al., 2018), where prey fish distributions are also concentrated (Litz et al., 2013; Phillips et al., 2017). Variation in Columbia River plume circulation and the formation of convergent fronts occur at temporal periods of hours to days (Jay, Pan, Orton, & Horner‐Devine, 2009; Jay, Zaron, & Pan, 2010), which is often not detectable at the sampling resolution of the ship surveys. Aerial surveys may have occurred during a period when Columbia River plume circulation or prey distributions caused lower densities of murres near Willapa Bay and Grays Harbor compared with oceanographic conditions when the ship surveys occurred. Based on our observations, the area to the north and south of the Columbia River mouth is a high‐use area for all murres in this study, although fine‐scale variation in distributions and changes throughout the study period suggest that different groups of birds may use this habitat differently during the spring and summer.

The use of satellite telemetry in this study offered the opportunity to expand the spatial extent and resolution by recording near‐continuous information about each individual murre's location, thus eliminating sampling constraints imposed by predetermined ship or aerial transects. This enabled us to demonstrate that the spatial extent of individual murres during the breeding season can encompass nearshore waters of California, Oregon, Washington, and British Columbia, essentially the full range of the California Current. Tags transmitted continuously for an average of two months between May and July, so we attained more continuous sampling of murre distributions compared with discrete ship and aerial surveys. Because we captured birds at sea, rather than at a colony, breeding status prior to tagging is unknown. There were no major differences in the sex ratio of tagged murres and their movement patterns, suggesting a somewhat random sample, but whether murres segregate at sea in relation to age or breeding status, or colony of origin, is unknown. Future research on this topic would provide important insight on murre conservation and management in the northern California Current (Thomas & Lyons, 2017). Tracking a small number of individuals can lead to large variability in observed habitat use (Fossette et al., 2014; Hays et al., 2016; Lindberg & Walker, 2007), and sample size may have also influenced the observed results. Of the 12 murres tagged, one flew to California, and five flew to Canada. To better understand the spatial and temporal extent of tagged animal distributions, Lindberg and Walker (2007) used simulations to estimate that at least 20–30 tagged individuals were necessary to reveal population patterns. Increasing the number of individual tag deployments may also provide better insight into comparability of different perspectives and platforms used to evaluate animal distributions.

The choice of survey perspective, platform, and spatiotemporal extent can be influenced by study objectives, accessibility of the area, sampling logistics, and available resources (Ainley et al., 2012). This research demonstrates that for surveys where objectives include obtaining accurate population abundance estimates and spatial use of coastal areas by a large‐bodied seabird, an Eulerian perspective using either ship or aerial survey methods produces similar results, although the spatial extent of survey transects can limit inferences on a population's full spatial extent (see also Briggs et al., 1985b, Henkel et al., 2007). Ships are ideal platforms to sample concurrent abiotic and/or biotic parameters such as sea surface temperature and prey density (Ainley et al., 2012), and therefore offer potentially more opportunities for ecological studies. Aerial surveys, however, can accomplish a survey in a much smaller amount of time, are not as limited by sea surface conditions and ocean depth, and may capture higher‐resolution variation in density distributions. In comparison, a Lagrangian perspective using satellite telemetry enables a much larger spatiotemporal sampling range compared to Eulerian surveys, allowing for a more extensive analysis of habitat use throughout a seabird's potential range. However, these results demonstrate that data from satellite telemetry of birds captured and tagged at sea may not be representative of the full population of interest (Priddel et al., 2014), and space use may not be necessarily related to actual density at sea (Ainley et al., 2012). By collecting and comparing concurrent data from three independent platforms, we obtained a more comprehensive understanding of the distribution of the murre population during the breeding season in the northern California Current, including connectivity to populations in British Columbia and California.

While ship‐based, aerial, and telemetry surveys can provide complementary information on species distributions, the results of this study indicate that a thorough assessment of the spatial extent and synopticity of relevant data is an important first step before integrating methodological perspectives. Depending on a study's objective, the spatiotemporal mismatch between independent data sets may bias observed species' distributions and relationships to habitat features. For example, a comparison between a ship‐based (Santora, Ralston, & Sydeman, 2011) and a telemetry‐based (Adams et al., 2012) survey of sooty shearwater (*Ardenna grisea*) distributions off the central California coast demonstrated that shearwaters observed from either perspective use the same general habitat, but that tagged birds were concentrated nearshore where larger vessels could not survey (c.f., Watanuki et al., 2016). Whether observed differences in spatial distributions of shearwaters were related solely to a spatiotemporal mismatch in sampling coverage, or possibly to differential habitat use or prey availability, remains unknown.

Efforts to combine Eulerian and Lagrangian perspectives using seabird counts within quantitative models have been conducted (Hyrenbach, Keiper, Allen, Ainley, & Anderson, 2006; Louzao et al., 2009; Yamamoto et al., 2015), and methods continue to be refined (Watanuki et al., 2016). Development of separate habitat models using data from each sampling perspective, and then comparing and integrating results across models, presents a powerful tool to quantify factors influencing marine mammal and seabird distributions and habitat use (Watanuki et al., 2016). This integrative approach has facilitated ongoing efforts to identify and delineate marine protected areas for multiple mobile marine predators (Ballard, Jongsomjit, Veloz, & Ainley, 2012; Camphuysen, Shamoun‐Baranes, Bouten, & Garthe, 2012; Perrow et al., 2015), as well as dynamic ocean management approaches (Hazen et al., 2016,2018; Maxwell et al., 2015). This type of habitat modeling could be a useful next step for the data presented here, especially in combination with Eulerian survey data from areas used by tagged murres in California and British Columbia to provide a comprehensive analysis of common murre spatial distributions along the west coast. Given the expected growth of telemetry studies (Hart & Hyrenbach, 2009) and efforts to integrate independent data sets (Watanuki et al., 2016), our results serve as a case study on how sampling perspective and choice of platform can influence spatiotemporal observations of species distributions.

## CONFLICT OF INTEREST

None declared.

## AUTHOR CONTRIBUTIONS

This study was conceived and designed by EMP, JA, and JEZ. Data were collected by EMP, JA, JEZ, and JJF. EMP conducted data analyses with assistance from JA, JEZ, JJF, and JKH. JA, JEZ, JJF, and JKH contributed to writing the manuscript with EMP. EMP edited the final manuscript, and all authors approve of its submission.

## Data Availability

Data supporting this manuscript have been deposited in Dryad https://doi.org/10.5061/dryad.1hg2n56.
